# Retractions in Dermatology Literature Between 1982 and 2022: Cross-sectional Study

**DOI:** 10.2196/39021

**Published:** 2023-03-28

**Authors:** Austin Huang, Kevin Y Huang, Soo Jung Kim

**Affiliations:** 1 Department of Dermatology Baylor College of Medicine Houston, TX United States; 2 Department of Molecular Biosciences The University of Texas at Austin Austin, TX United States

**Keywords:** publication, retraction, bibliometrics, dermatology

The recent growth in the number of dermatology publications, as well as the increasing rate of retractions in other fields of medicine, has raised questions about how the field of dermatology compares in terms of this metric [1,2]. In this study, we evaluated retracted publications in the field of dermatology and explored the trends of retraction over the past four decades.

All retracted dermatology-related articles from 1982 to 2022 were identified on the Retraction Watch Database. The Retraction Watch Database, compiled and maintained by the Retraction Watch team, is the largest searchable database of retracted scientific articles publicly available to researchers [3]. Information regarding article type, country of authors, reasons for retraction, publication year, and the number of months between publication and retraction for each paper were collected, and linear regression was performed to assess trends of retractions over time.

Between 1982 and 2022, there were a total of 178 retracted articles in the field of dermatology. The most common article types were “Research Article” (n=91), “Review Article” (n=31), “Clinical Study” (n=25), and “Case Report” (n=21). The majority of these papers originated from China (n=33), the United States (n=32), the United Kingdom (n=20), India (n=19), and South Korea (n=16). The most frequent reasons given for retraction included “Errors in Analyses, Data, Image, Materials, Methods, Text, Results, or Conclusions” (n=46) and “Duplication of Article, Data, Image, or Text” (n=45). Eight articles were retracted due to falsification or fabrication of data and results. Linear regression determined a moderate negative correlation between the year of publication and the number of months between publication and retraction, with *P*<.001 and multiple *R*^2^=0.48 (Figure 1).

Consistent with the findings in other fields [2,4], these results reveal that the absolute number of retracted dermatology publications has markedly increased over the past two decades (Figure 2). The exact reason for this phenomenon is unclear, whether it is due to an increase in the number of dermatology publications, an increase in the rate of duplications submitted by authors, or a greater vigilance by journals to identify reasons for potential retraction. However, it appears that more recent dermatology publications have been undergoing the process of retraction significantly quicker than older papers. The negative association observed between the year of publication and the time between publication and retraction indicates that the latter rationale may contribute the most to this occurrence.

While there is no evidence to suggest that the increase in the number of retractions in dermatology has been accompanied by an increase in the output of low-quality research, the mantra of “publish-or-perish” is frequently discussed among academics concerning the field of medicine in general [5]. Despite the pressure to publish from their institution, their colleagues, or their own self-interest, authors must continue to accurately analyze data and adhere to ethical research guidelines, as it appears that most retractions occur due to errors rather than falsifications and fabrications. Similarly, journal staff members should continue to diligently monitor the articles for potential issues that may warrant retraction. Limitations to this study include the reliance on the Retraction Watch Database to identify retracted publications in the field of dermatology, which may have resulted in unidentified articles relevant to this analysis. Our study naturally does not account for manuscripts that were rejected during the peer-review process due to errors, duplications, and falsifications; however, observing the trend in retractions of published papers in the dermatology literature benefits researchers and journal editors alike.

**Figure 1:**
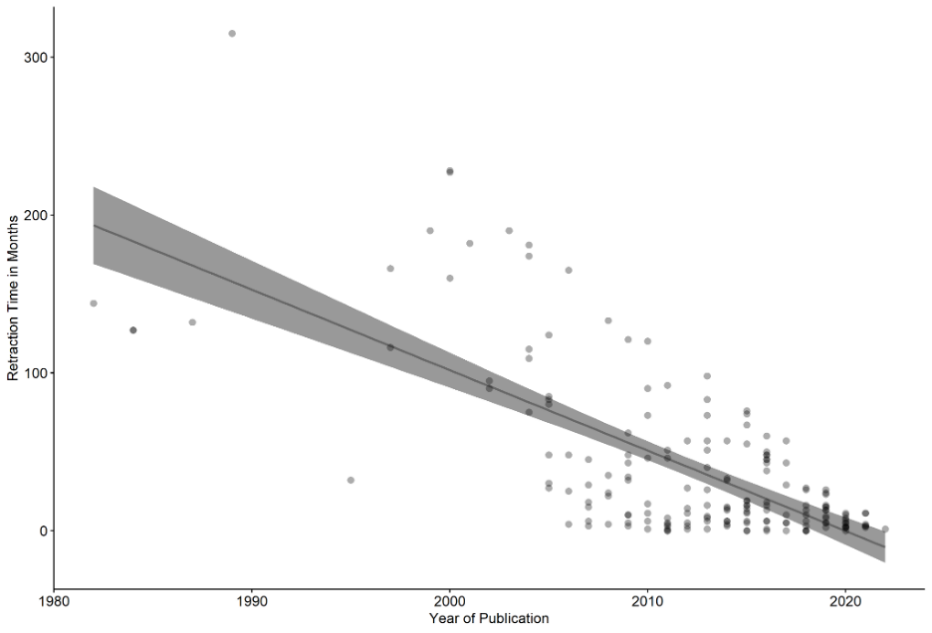
Scatter plot depicting the moderate negative association between the year of publication and retraction time in months. *P*<.001 and multiple *R*^2^=0.48.

**Figure 2:**
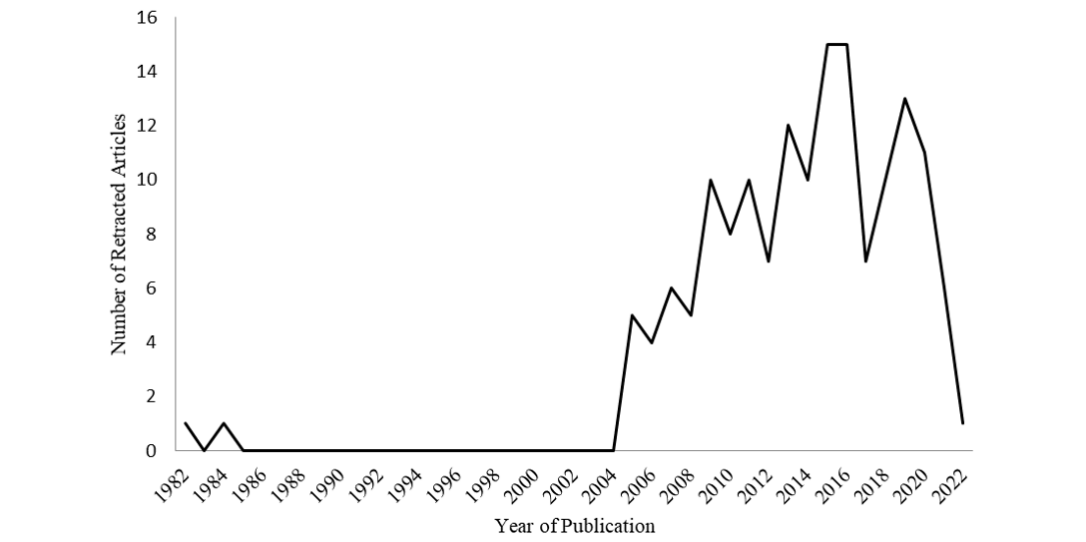
Line chart depicting the increase in the number of retracted articles in the dermatology literature over the past two decades.
